# Comparative Evaluation of the Bone Regenerative Potential of a Novel Calcium Silicate-Modified Calcium Carbonate Graft Material: Histological and Micro-Computed Tomography Assessment Using a Rat Calvarial Defect Model

**DOI:** 10.3390/jfb16090337

**Published:** 2025-09-09

**Authors:** Masataka Nakayama, Yu Kataoka, Naoki Kitamura, Chie Watanabe, Satoko Kujiraoka, Kikue Yamaguchi, Yuma Seki, Toshitake Furusawa, Hidero Unuma, Motohiro Munakata

**Affiliations:** 1 Department of Implant Dentistry, Showa Medical University Graduate School of Dentistry, 2-1-1, Kita-senzoku, Ota-ku, Tokyo 145-8515, Japan; masa.nakayama@dent.showa-u.ac.jp (M.N.); gd22-n008@dent.showa-u.ac.jp (N.K.); kyamaguchi@dent.showa-u.ac.jp (K.Y.); firi@amber.plala.or.jp (T.F.); munakata@dent.showa-u.ac.jp (M.M.); 2 Department of Biomaterials and Engineering, Showa Medical University Graduate School of Dentistry, 1-5-8, Hatanodai, Shinagawa-ku, Tokyo 142-8555, Japan; chie@dent.showa-u.ac.jp; 3 Department of Dental Education, Showa Medical University Graduate School of Dentistry, 1-5-8, Hatanodai, Shinagawa-ku, Tokyo 142-8555, Japan; 4 Tsurumi University Dental Hospital, 2-1-3 Tsurumi, Tsurumi-ku, Yokohama-shi, Kanagawa 230-8501, Japan; kujiraoka-s@tsurumi-u.ac.jp; 5 Graduate School of Science and Engineering, Yamagata University, 4-3-16 Jonan, Yonezawa City, Yamagata 992-8510, Japanunuma@yz.yamagata-u.ac.jp (H.U.)

**Keywords:** bone graft materials, calcium carbonate, β-tricalcium phosphate, calcium silicate-modified calcium carbonate, new bone formation, dental implant, bone graft

## Abstract

In the present study, we evaluated the usefulness of a porous sintered calcium carbonate body with CaSiO_3_ by comparing its osteogenic capacity with that of calcium carbonate without CaSiO_3_ and that of β-tricalcium phosphate (TP). A cranial defect model of eight-week-old male Wistar rats was divided into three groups: calcium carbonate (CC), calcium carbonate-CaSiO_3_ composite (CC+CS), and TP. Micro-computed tomography (CT) and histological analysis were performed at four and eight weeks postoperatively. Upon quantitative evaluation of newly formed bone volume by radiography, the CC+CS group demonstrated the highest value at eight weeks postoperatively and exhibited significantly more new bone formations than the CC group (*p* < 0.05). Upon histological evaluation, the CC+CS group demonstrated significantly higher new bone formation than the CC group (*p* < 0.05). Furthermore, in terms of residual graft material ratio, at eight weeks postoperatively, the amount of residual graft material in the CC+CS group was significantly higher than that in the TP group (*p* < 0.05). Therefore, the addition of CaSiO_3_ enhances the functional regulation of calcium carbonate-based artificial bone and can be incorporated in bone graft materials.

## 1. Introduction

In recent years, with the widespread adoption of implant treatment, its indications have expanded, and various bone augmentation techniques, such as vertical bone augmentation [1,2,3] and horizontal(lateral) bone augmentation with guided bone regeneration (GBR) [4,5], sinus augmentation [6,7], and alveolar ridge preservation [8], have become commonly used in clinical practice. These procedures have demonstrated high success rates and long-term implant survival rates in multiple systematic reviews and randomised controlled trials.

The bone graft materials used for bone augmentation include autografts, allografts, xenografts, and synthetic bone substitutes, each with their own advantages and challenges [9]. Although autogenous bone is considered the ‘gold standard’, facilitating osteogenesis, osteoinduction, and osteoconduction, it has certain limitations, such as limited harvestable quantity, donor site invasion, infection risk, and prolonged surgical time [10]. Allografts possess an osteoinductive capacity but face challenges of infection risk and ethical issues. Xenografts, especially those derived from bovine sources, exhibit excellent osteoconductivity; however, due to poor bioresorbability and prolonged persistence in the body, have reportedly result in reduced bone-to-implant contact rate and increased infection risk [11].

Several artificial bone graft materials, such as calcium phosphate-based materials (hydroxyapatite, β-tricalcium phosphate [β-TCP], and octacalcium phosphate), carbonate apatite, and polymer composites, have been widely developed as materials with excellent biocompatibility and osteoconductivity. Synthetic bone materials reduce the risk of infection and preclude ethical concerns; although, they possess the advantage of stable bone formation, they lack osteoinductive and osteogenic properties, and the low volume of newly formed bone remains an issue [12]. Therefore, in implant treatments, the development of artificial bone materials that leave little residual graft and generate large volumes of new bone is critical.

Calcium carbonate has long been used as a bone graft material owing to its high biocompatibility and osteoconductivity. In particular, the exoskeleton of natural coral, mainly composed of calcium carbonate and having a three-dimensional porous structure similar to cancellous bone, has been studied extensively as a bone graft material since the 1970s. The exoskeleton of madrepore corals, such as porites (a type of stony coral), has mechanical properties similar to bone, with good biocompatibility, osteoconductivity, and biodegradability. While coral itself does not possess osteoinductive properties, it functions as a carrier for growth factors and a scaffold for cells and can be used as a bone graft material by appropriately controlling the resorption rate [13].

However, natural coral presents significant challenges in controlling the resorption rate, adjusting porosity and pore size, and is limited by the species that can be used, environmental concerns related to the ocean, and cost; therefore, research has progressed focusing on alternative synthetic sintered calcium carbonate materials [12,13,14,15]. Traditionally, calcium carbonate is prone to thermal decomposition, making sintering challenging. However, Umemoto et al. demonstrated that sintering is possible under a one-atmosphere CO_2_ environment, and porous sintered bodies created in this way have demonstrated potential for bone regeneration in vivo. Furthermore, adding silica (SiO_2_) reportedly improved mechanical strength, resulting in favourable bone formation in a rat calvarial defect model [14,15]. Recently, the modification of calcium carbonate-based materials has advanced through the addition of inorganic components that promote bone formation, such as SiO_2_ and β-wollastonite (β-CaSiO_3_), and comparative studies with ceramics containing β-TCP or wollastonite are underway [16,17]. Xu et al. [ used a rabbit calvarial defect model to compare the in vivo bone regeneration and resorbability of porous β-CaSiO_3_ ceramics with similarly structured porous β-TCP ceramics. In the study, β-CaSiO_3_ demonstrated significantly faster resorption and more prominent new bone formation than β-TCP, suggesting that β-CaSiO_3_ is a useful biodegradable material that can promote bone regeneration [18]. In addition, Barbosa et al. fabricated porous composite ceramics by mixing β-CaSiO_3_ and β-TCP in different ratios and evaluated their biocompatibility and bone regenerative potential in a rabbit femoral defect model. They reported that none of the composite materials exhibited cytotoxicity and that they promoted bone formation during the early stages of the bone healing process. Umemoto et al. demonstrated that CaCO_3_ significantly promotes bone formation compared to β-TCP and also results in significantly less fibrous tissue formation. Barbosa et al. investigated the in vivo behaviour of β-TCP/CaSiO_3_ materials and suggested that CaSiO_3_ may be more suitable than β-TCP as a matrix for dispersing CaSiO_3_. CaCO_3_ sintered bodies are a promising material recently reported by Umemoto et al., and their potential to enhance the performance of bone graft materials through composite formulations with other substances such as CaSiO_3_ has been recognised. Therefore, this study initiated an evaluation of the performance of CaCO_3_/CaSiO_3_ composites [19].

In the present study, we sought to determine the usefulness of the addition of a silicate to bone graft materials and the efficacy of calcium carbonate with added CaSiO_3_ by comparing the osteogenic potential of porous sintered calcium carbonate and calcium carbonate-calcium silicate composite with β-TCP, with similar porosity and pore size, through radiographic analysis using micro-computed tomography (CT) and histological evaluation.

## 2. Materials and Methods

### 2.1. Biomaterials

**Figure 1:**
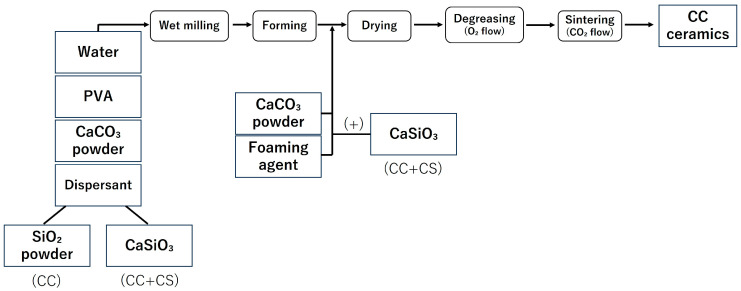
Sample preparation flow (CC: calcium carbonate containing silica, CC+CS: calcium carbonate-calcium silicate composite).

**Figure 2:**
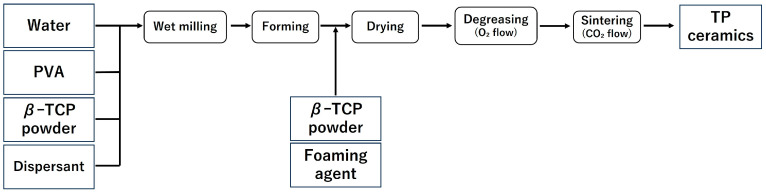
Sample preparation flow (TP: β-tricalcium phosphate).

In this study, the samples were fabricated in accordance with the report by Umemoto et al. [14,15]. The fabrication process of the samples is shown in Figure 1 and Figure 2, and the list of reagents used is shown in Table 1 and Table 2.

#### 2.1.1. Manufacture of Calcium Carbonate Containing Silica (CC)

Samples ① to ⑤,shown in Table 1, were pulverised in a ball mill for 24 h and adjusted into a slurry state. After the ball milling process, the slurry was separated from the balls using a wire mesh, and the yield of the obtained slurry was measured. Subsequently, a foaming agent was added to the collected slurry to generate sufficient foaming, and high-purity calcium carbonate powder was added to stabilise the foam of the foamed slurry. The resulting foamed slurry was poured into moulds (4 cm × 4 cm × 5 cm) made of plastic plates, frozen for 1 h using liquid nitrogen vapour, and then dried for 24 h. The resulting freeze-dried body was heat treated in an oxygen atmosphere, first by raising the temperature to 150 °C over 3 h, and then holding at 530 °C for 7 h to perform defatting. After defatting, the sample was heated in a carbon dioxide atmosphere at 10 °C/min and sintered at 850 °C for 1 h to obtain a block-shaped sintered body (CC).

#### 2.1.2. Production of β-TCP (TP)

Samples ① to ④ listed in Table 2 were pulverised using a ball mill for 24 h to prepare a slurry. After pulverisation, to prevent the phase transition from β-TCP to α-TCP, magnesium oxide (MgO) (0.3 g) was added, and the mixture was further milled for 1 h. After the second milling, the balls and slurry were separated using a wire mesh, and the yield of the obtained slurry was measured. Subsequently, a foaming agent was added to the collected slurry and foamed, followed by the addition of β-TCP powder (5 g) to stabilise the formed foam.

Following the same procedure as with CC, freeze-drying, defatting, and sintering were carried out, and a block-shaped sintered body (TP) was obtained (Figure 2).

#### 2.1.3. Production of Calcium Carbonate-Calcium Silicate Composite (CC+CS)

In addition to samples ① to ④ in Table 1, calcium silicate (CaSiO_3_) powder (4.5 g) was added. Similarly to the CC group, a foaming agent was added to the slurry, and high-purity calcium carbonate powder and CaSiO_3_ powder (1 g) were added to stabilise the generated foam.

Thereafter, similar to the CC group, freeze-drying, defatting, and sintering were performed to obtain a block-shaped sintered body (CC+CS).

#### 2.1.4. Production of Calcium Silicate (CaSiO_3_) (CS)

A solution of 59.3 g (0.25 mol) of calcium nitrate tetrahydrate (Ca(NO_3_)_2_·4H_2_O) and 71.05 g (0.25 mol) of sodium metasilicate nonahydrate (Na_2_SiO_3_·9H_2_O) was mixed, and centrifugation was performed for a total of four times.

The collected precipitate was dried at 120 °C for 12 h and then pulverised into powder. The resulting powder was sintered at 1200 °C for 2 h to produce α-CaSiO_3_.

#### 2.1.5. Manufacturing of Implant Fillers

The block-shaped sintered bodies CC, CC+CS, and TP were shaped into cylinders with a diameter of 5 mm and sliced with a scalpel into 1 mm thick discs, resulting in cylindrical implant materials of CC, CC+CS, and TP with 1 mm thickness. The porosity was adjusted during the foaming of the slurry and was standardised to 73% (±3%) for the CC and CC+CS implant materials, and 74% (±4%) for the TP implant material.

**Figure 3:**
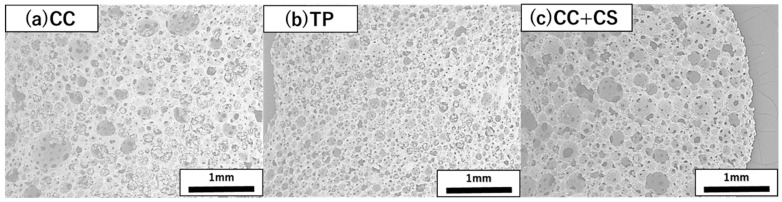
Representative scanning electron microscopy images of the cross-sections of three novel bone graft materials (25×): (**a**) CC, (**b**) TP, and (**c**) CC+CS. (CC: calcium carbonate containing silica, TP: β-tricalcium phosphate, CC+CS: calcium carbonate-calcium silicate composite).

Scanning electron microscopy (SEM) images of each implant material (CC, TP, and CC+CS) were obtained using a Hitachi TM3000 SEM (Hitachi High-Technologies Corporation, Tokyo, Japan) and are shown in Figure 3. Powder X-ray diffraction (XRD) analysis was performed using a diffractometer (Multiflex; Rigaku Corporation, Tokyo, Japan), which showed that CaSiO_3_ added to CaCO_3_ existed in the form of Ca_3_Si_2_O_7_ in the sintered body (data not shown).

### 2.2. Study Groups and Surgical Procedure

All in vivo experiments were performed in accordance with the guidelines and with the approval of the Animal Experimentation Committee of Showa Medical University (Approval No. 15067, approved in March 2023).

The surgical procedures were performed at the Laboratory Animal Centre of Showa medical University (Tokyo, Japan) using male Wistar rats weighing 250–270 g. Thirty male Wister rats, 8 weeks of age, were used in this study. Animal experiments were conducted under six different experimental conditions depending on the implantation material and the period of implantation, and the number of samples for each condition was 5.

A total of 30 rats were randomly assigned to three equal groups according to the type of biomaterial used:

- Group 1: CC group
- Group 2: TP group
- Group 3: CC+CS group

Following induction of anaesthesia with inhaled isoflurane, a mixture of medetomidine (0. 15 mg/kg), midazolam (2 mg/kg), and butorphanol (2.5 mg/kg), diluted with phosphate-buffered saline (1.45 mL/kg) to a final administration volume of 2.5 mL/kg, was administered subcutaneously using a 27-gauge needle. All the procedures were conducted with utmost consideration aimed towards minimising animal pain and distress.

**Figure 4:**
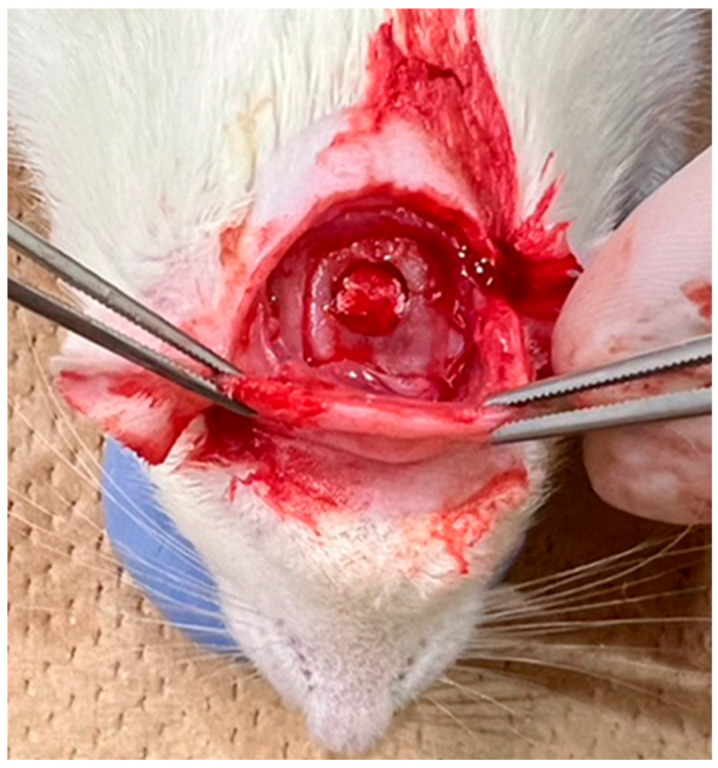
Filling the skull defect in a rat with a filling material.

After shaving the surgical site, disinfection was performed with iodine. A transverse skin incision of approximately 3 cm was made using scissors along the line connecting the base of both ears, followed by subcutaneous dissection to expose the periosteum. The periosteum was incised in a U-shape using a No. 15 scalpel to expose the parietal bone. Under saline cooling, a bone defect approximately 5 mm in diameter was created in the centre of the parietal region using a trephine bur (inner diameter: 4.8 mm; outer diameter: 5.8 mm; Helmut Zepf Medizintechnik GmbH, Seitingen-Oberflacht, Germany), as shown in Figure 4. The materials from each group were implanted into this defect. The surgical site was closed using single interrupted sutures (NYLON 5-0 c-13; CROUNJUN, Kono Seisakusho Co., Ltd., Chiba, Japan). Euthanasia was performed at 4 and 8 weeks after implantation by CO_2_ overdose, and the skulls were harvested.

### 2.3. Micro-CT Analysis

Micro-CT analysis was performed using horizontal sections of the skull samples. An X-ray CT scanner (ScanXmate-L090H; Comscan, Kanagawa, Japan) was used with the following settings: tube voltage, 80 kV; tube current, 83 µA; scan time, 30 s; slice interval, 0.16 mm; and slice thickness, 0.16 mm. Micro-CT images were reconstructed using image software (coneCTexpress v1.59; White Rabbit Co. Ltd., Tokyo, Japan). Quantitative analysis was subsequently conducted using three-dimensional bone morphometry software (TRI/3D-BON-FCS64; RATOC Systems, Inc., Tokyo, Japan) and image analysis software (WinROOF Education, Mitani Corporation, Tokyo, Japan; Version 2023) to calculate the percentage of new bone formation and residual graft material for each group.

### 2.4. Histological Analysis

Histological analysis was performed using sagittal sections of the samples. Samples collected from the experimental sites of the CC, CC+CS, and TP groups were immersed in 4% buffered formalin at room temperature for 2 days and then embedded in paraffin. Serial longitudinal sections of approximately 5 μm were prepared and stained with hematoxylin and eosin (HE) to distinguish tissue types. Observation was conducted using an optical microscope (CKX41, Olympus, Tokyo, Japan). Image analysis software (WinROOF Education, Mitani Corporation, Tokyo, Japan; Version 2023) was used to calculate the percentage of new bone formation (%) and residual graft material (%) for each group.

### 2.5. Statistical Analysis

The statistical analysis of the percentages of new bone formation and residual graft material was performed using analysis of variance and the Student–Newman–Keuls test, with a significance level of *p* < 0.05.

## 3. Results

### 3.1. Results of Micro-CT

**Figure 5:**
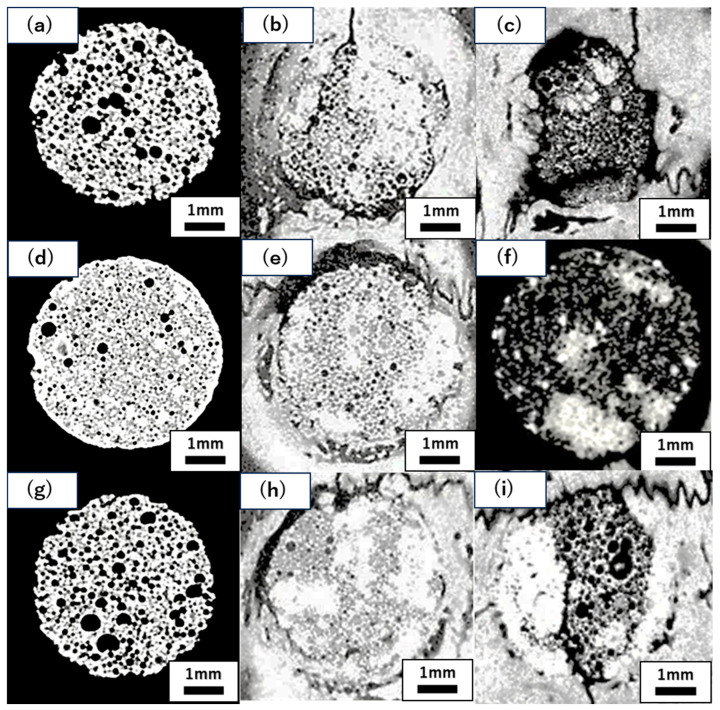
Representative micro-CT images of CC, TP, and CC+CS groups at pre-implantation, four and eight weeks. (**a**) Pre-implantation (CC), (**b**) four weeks post-operation (CC), (**c**) eight weeks post-operation (CC); (**d**) pre-implantation (TP), (**e**) four weeks post-operation (TP), (**f**) eight weeks post-operation (TP); (**g**) pre-implantation (CC+CS), (**h**) four weeks post-operation (CC+CS), (**i**) eight weeks post-operation (CC+CS). (CC: calcium carbonate, TP: β-tricalcium phosphate, CC+CS: calcium carbonate-calcium silicate composite).

**Figure 6:**
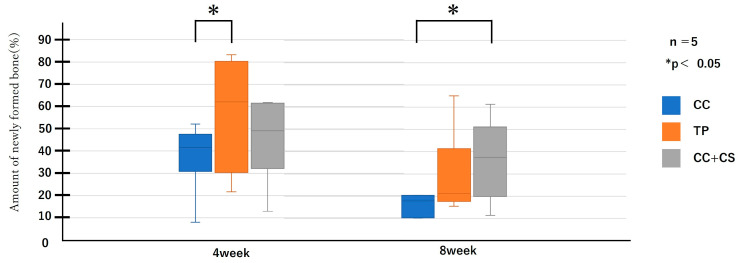
Micro-computed tomography analysis of newly formed volume (CC: calcium carbonate containing silica, TP: β-tricalcium phosphate, CC+CS: calcium carbonate-calcium silicate composite).

**Figure 7:**
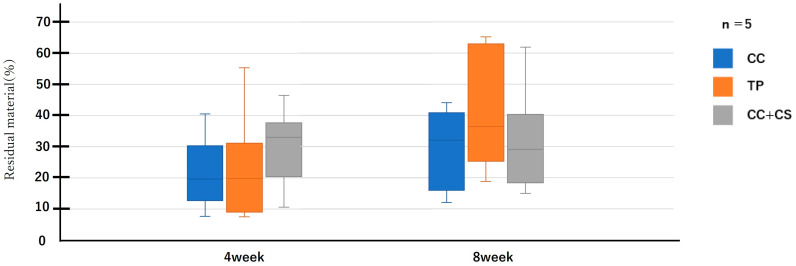
Micro-computed tomography analysis of the residual graft volume (CC: calcium carbonate containing silica, TP: β-tricalcium phosphate, CC+CS: calcium carbonate-calcium silicate composite).

Micro-CT images before graft material placement, and at four and eight weeks post-surgery for each group are shown in Figure 5. Further image analysis quantifying new bone volume and remaining graft material are shown in Figure 6 and Figure 7.

The micro-CT image analysis revealed clear differences among groups in terms of the structural changes in the graft material and progression of new bone formation. Upon comparing the CC and TP groups, high-density areas extensively reaching the centre of the defect were observed in the TP group at all time points, indicating more pronounced progression of new bone formation compared to that in the CC group. Particularly at eight weeks post-surgery, the TP group exhibited a homogeneous high-density structure spreading from around the graft material to the centre of the defect, suggesting a higher degree of bone formation than the CC group. Next, comparing the CC group and the CC+CS group, a clear bone formation-promoting effect of CS was observed. By four weeks post-surgery, the CC+CS group already exhibited greyish high-density areas throughout the entire graft material, indicating earlier progression of bone formation than the CC group. At eight weeks post-surgery, the CC+CS group exhibited a more extensive and denser bone-like structure. Notably, uniform bone formation progressed even into the centre of the defect, suggesting a marked bone-inductive effect of CS.

Quantitative evaluation of new bone volume by micro-CT revealed that, at four weeks post-surgery, the TP group had the highest density and the difference was significant when compared with that of the CC group (*p* < 0.05). However, at eight weeks, the CC+CS group exhibited the highest density, significantly exceeding the CC group (*p* < 0.05). The TP group tended to show higher density than the CC group at eight weeks, but no statistically significant difference was found. Meanwhile, no significant differences were observed in the percentage of remaining graft material at either four or eight weeks.

Quantification of newly formed bone and residual graft volume in the calvarial defect area is shown in Figure 6 and Figure 7, respectively. At four weeks, the TP group showed significantly greater new bone formation than the CC group (* *p* < 0.05). At eight weeks, the CC+CS group exhibited significantly higher new bone formation than the CC group (* *p* < 0.05). No significant differences in the residual graft volume were observed among the groups at either time point; however, the TP group tended to retain more graft at eight weeks.

Data are presented as box-and-whisker plots (min–max, with median and mean). Blue: CC, Orange: TP, Grey: CC+CS.

### 3.2. Results of Histological Analysis of the Samples

**Figure 8:**
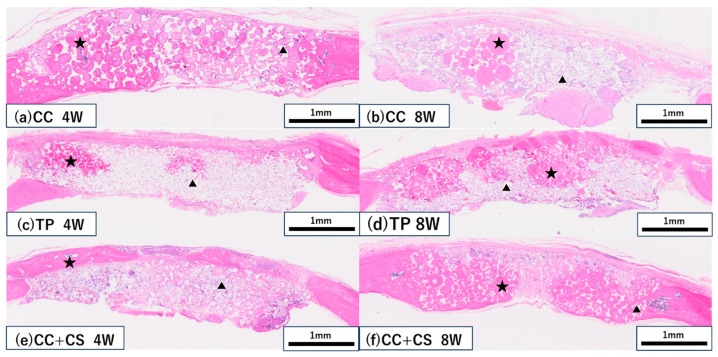
Histological images of the grafted sites (haematoxylin and eosin staining). (**a**,**b**) CC group at four and eight weeks, respectively; (**c**,**d**) TP group at four and eight weeks, respectively; (**e**,**f**) CC+CS group at four and eight weeks, respectively. (CC: calcium carbonate, TP: β-tricalcium phosphate, CC+CS: calcium carbonate-calcium silicate composite). ★ New bone, ▲ Residual material.

**Figure 9:**
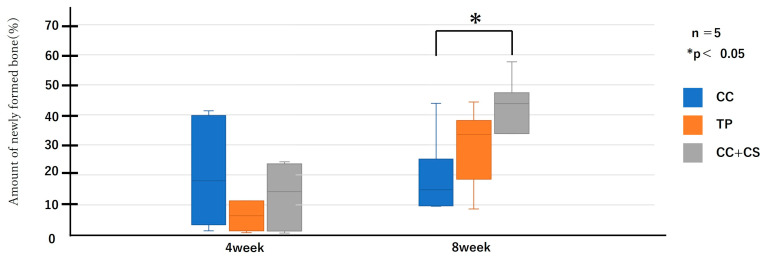
Histological analysis of newly formed formation (CC: calcium carbonate containing silica, TP: β-tricalcium phosphate, CC+CS: calcium carbonate-calcium silicate composite).

**Figure 10:**
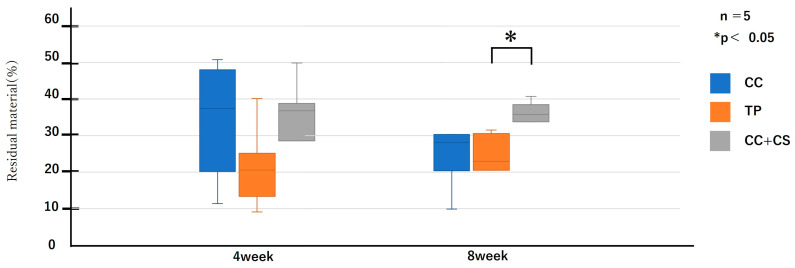
Histological analysis of residual bone volume (CC: calcium carbonate containing silica, TP: β-tricalcium phosphate, CC+CS: calcium carbonate-calcium silicate composite).

Histological evaluation was performed across the entire graft area (5 × 1 mm) at four and eight weeks post-surgery. Representative histological images are shown in Figure 8, and the quantified results of new bone volume and remaining graft material from the image analysis are shown in Figure 9 and Figure 10.

In both the CC and TP groups, new bone formation was confirmed at eight weeks post-surgery, but more pronounced bone formation was observed in the CC+CS group. In the CC group, fibrous tissue predominated at four weeks, with an increase in new bone confirmed by eight weeks. In contrast, the CC+CS group demonstrated clear bone formation already at four weeks, and by eight weeks, mature bone-like tissue and bone marrow cavity-like structures were widely observed. In the TP group, many graft materials remained at four weeks with limited bone formation; however, by eight weeks, bone formation progressed alongside graft material resorption. These results suggest that the addition of silicon may contribute to promoting bone formation in both calcium carbonate- and β-TCP-based materials.

As shown in Figure 9 and Figure 10, the comparison of new bone formation amounts from histological analysis revealed no significant differences among the four groups at four weeks post-surgery. However, at eight weeks, the CC+CS group exhibited significantly more new bone formation than the CC group (*p* < 0.05). These findings indicate particularly prominent bone formation in the CC+CS group at eight weeks. Additionally, evaluation of the remaining graft material revealed no significant differences among the groups at four weeks, but at eight weeks, the CC+CS group exhibited a significantly higher amount of remaining graft material than the TP group (*p* < 0.05).

Quantification of newly formed bone and residual graft material based on sagittal histological sections is shown in Figure 9 and Figure 10, respectively. At eight weeks, the CC+CS group exhibited significantly greater new bone formation than the CC group (*p < 0.05). Additionally, the residual graft amount in the TP group was significantly lower than that in the CC+CS group at eight weeks (*p < 0.05), suggesting faster resorption.

Data are presented as box-and-whisker plots (min–max, with median and mean). Blue: CC, Orange: TP, Grey: CC+CS.

## 4. Discussion

In the present study, we demonstrated that the CC+CS group exhibited the most effective outcomes in both the micro-CT and histological analyses. These findings indicate that the bone regeneration effect of artificial bone graft material containing silicon is effective. β-TCP is a calcium phosphate-based material with excellent osteoconductivity and moderate bioresorbability, enabling balanced bone substitution with newly formed bone. Furthermore, the Ca^2+^ and PO_4_^3−^ ions released during its degradation are known to promote osteoblast differentiation and extracellular matrix mineralisation, creating an environment suitable for bone formation [20,21]. Sustained Ca^2+^ release from β-TCP also reportedly contributes to the migration and differentiation of mesenchymal stem cells (MSCs), as well as to the upregulation of bone-related genes such as Runx2 and OCN [15,22]. In the present study, the TP group demonstrated the most pronounced bone formation at four weeks post-surgery compared to the CC group, which could be attributed to MSC recruitment and enhanced angiogenesis accelerating bone regeneration.

On the other hand, in the CC group, rapid early resorption of the graft material was observed histologically, with bone formation lagging behind the resorption rate. This finding is consistent with that of a previous report that demonstrated that highly porous natural coral-derived calcium carbonate exhibits high resorption rates that can disrupt the bone formation equilibrium [23]. Umemoto et al. also reported that, while highly pure sintered calcium carbonate shows excellent bioresorbability, the extent of bone formation is strongly influenced by the material’s resorption speed [14,15].

In the present study, although micro-CT analysis revealed less bone formation in the CC group, histological analysis confirmed partial bone formation within the graft material. This discrepancy may be explained by the fact that micro-CT primarily reflects the density of mineralised tissue, whereas histological sections can detect unmineralised bone matrixes and osteoblasts. Differences in the observation planes (horizontal section for micro-CT vs. sagittal section for histology) may also have contributed to the variation in the results. These findings suggest that micro-CT and histological analyses provide complementary information, and their combined use is effective for accurate understanding of bone regeneration.

Furthermore, the improved bone formation ability in the CC+CS group containing Si may be attributed to its ability to promote osteoblast proliferation and differentiation, and its upregulation of bone formation-related genes, such as ALP, Runx2, and OCN [24,25,26]. It is suggested that Si^4+^ ions enhance bone formation through the activation of BMP and Runx2 signalling cascades. This may contribute to an improved bone regeneration environment. Si also reportedly supports early bone formation processes by promoting collagen synthesis and extracellular matrix mineralisation. A review by Price et al. indicated that Si contributes to maintaining bone density and enhances bone formation, especially in postmenopausal osteoporosis [27]. In the present study, the CaSiO_3_ group (CC+CS group) revealed a tendency towards increased bone formation than the non-CaSiO_3_ group. Notably, the CC+CS group exhibited high-density new bone formation from four weeks, with the entire defect site replaced by bone tissue by eight weeks. These results presumably reflect the synergistic effect of CC’s osteoconductivity and Ca^2+^ release with Si’s molecular-level cell activation, which together establish a bone regeneration environment over time. Regarding the interaction between Si and Ca^2+^, several studies have reported their synergistic effects. Modi et al. reported that adding calcium glucoheptonate promoted osteoblast-like cell differentiation, and Aenglong et al. demonstrated that fish-derived calcium compounds strongly induced osteoblast differentiation in MC3T3-E1 cells [23,28]. Ni et al. demonstrated that high Ca^2+^ concentrations promoted MSC migration, proliferation, and osteopontin expression, supporting the important role of Si and Ca^2+^ interaction in bone regeneration [29,30]. However, a disadvantage of CaSiO_3_ is its strong alkalinity, which can raise local tissue pH and cause cytotoxicity if overdosed [31]. To address this, Barbosa et al. evaluated mixtures of CaSiO_3_ and β-TCP at various ratios, revealing that an optimal ratio avoids toxicity while maintaining biocompatibility and stimulating early bone repair [19].

Additionally, although the CC+CS group exhibited high bone formation, it also demonstrated a high amount of remaining graft material. The residual presence of CC+CS may contribute to improved mechanical stability during the early healing phase; however, excessive persistence could delay complete replacement by native bone, which warrants further investigation. Future studies are warranted to optimise the amount of Si to be added to achieve both high bone formation and low residual rates, investigate synergistic effects with other bioactive ions (Mg, Sr, Zn, etc.), and evaluate long-term resorption behaviour, bone-implant contact changes, and mechanical stability, which are important for clinical application.

## 5. Conclusions

- CC+CS demonstrated significantly greater bone formation than CC at eight weeks, as shown by both micro-CT and histology.
- The residual graft volume was higher in CC+CS than in TP.
- Adding CaSiO_3_ to calcium carbonate regulates bone formation and resorption.

## Figures and Tables

**Table 1:** Reagents used.

Reagent Name	(g)
① 99. 99% CaCO_3_ (Shiraishi Kogyo Co., Ltd., Osaka, Japan)	18
② 5% polyvinyl alcohol (PVA 500, Kanto Chemical Co., Inc., Tokyo, Japan)	4
③ Polycarboxylate polymer type surfactants (Poiz 520, Kao Corp., Tokyo, Japan) (Dispersant)	1
④ Distilled water	9
⑤ 20 wt% Silica Sol (SNOWTEX®-N, Nissan chemical, Tokyo, Japan)	0.53
1 wt% MIGHTY FA-S02, Kao, Tokyo, Japan (Foaming agent)	0.38
99. 99%CaCO_3_	5

**Table 2:** Reagents used.

Reagent Name	(g)
① β-Tricalcium phosphate (β-TCP), Taihei Chemical Industrial Co., Osaka, Japan	30
② 5%PVA (PVA 500, Kanto Chemical Co., Inc., Tokyo, Japan)	4
③ 1 wt% MIGHTY 3000S, Kao, Tokyo, Japan (Dispersant)	1
④ Distilled water	9
⑤ MgO (Kojundo chemical Laboratory Co., Ltd., Saitama, Japan)	0.3
1 wt% MIGHTY FA-S02(Foaming agent)	0.5
β-TCP	5

## Data Availability

The raw data supporting the conclusions of this article will be made available by the corresponding author upon request.

## Abbreviations

The following abbreviations are used in this manuscript: micro-CT: micro- computed tomography, β-TCP: β- Tricalcium phosphate, MSC: mesenchymal Stem Cells
